# Edge Effects on Foliar Stable Isotope Values in a Madagascan Tropical Dry Forest

**DOI:** 10.1371/journal.pone.0044538

**Published:** 2012-09-04

**Authors:** Brooke E. Crowley, Keriann C. McGoogan, Shawn M. Lehman

**Affiliations:** 1 Departments of Geology and Anthropology, University of Cincinnati Cincinnati, Ohio, United States of America; 2 Department of Anthropology, University of Toronto, Toronto, Ontario, Canada; KU Leuven, Belgium

## Abstract

Edge effects represent an inevitable and important consequence of habitat loss and fragmentation. These effects include changes in microclimate, solar radiation, or temperature. Such abiotic effects can, in turn, impact biotic factors. They can have a substantial impact on species, communities, and ecosystems. Here we examine clinal variations in stable carbon and nitrogen isotope values for trees along an edge-interior gradient in the dry deciduous forest at Ankarafantsika National Park. We predicted that soil respiration and differences in solar irradiance would result in stratified δ^13^C values where leaves collected close to the forest floor would have lower δ^13^C values than those growing higher up in the canopy. We also anticipated that plants growing at the savannah-forest boundary would have higher δ^13^C and δ^15^N values than plants growing in the forest interior. As expected, we detected a small but significant canopy effect. Leaves growing below 2 m from the forest floor exhibit δ^13^C values that are, on average, 1.1‰ lower than those growing above this threshold. We did not, however, find any relationship between foliar δ^13^C and distance from the edge. Unpredictably, we detected a striking positive relationship between foliar δ^15^N values and increasing distance into the forest interior. Variability in physiology among species, anthropogenic influence, organic input, and rooting depth cannot adequately explain this trend. Instead, this unexpected relationship most likely reflects decreasing nutrient or water availability, or a shift in N-sources with increasing distance from the savannah. Unlike most forest communities, the trees at Ampijoroa are growing in nutrient-limited sands. In addition to being nutrient poor, these well-drained soils likely decrease the amount of soil water available to forest vegetation. Continued research on plant responses to edge effects will improve our understanding of the conservation biology of forest ecosystems in Madagascar.

## Introduction

A fundamental issue in ecology and conservation biology is determining how edge effects influence plant communities [1]. Numerous studies have established that the structure and function of plant communities at forest edges differ from those in the relatively more homogenous interior forest and in the surrounding matrix [2], [3], [4]. This ecological relationship has been defined as edge effects or edge influence [5], [6]. Researchers have focused considerable effort on studying edge effects because forests are becoming increasingly fragmented in many parts of the world [7], which results in edge habitats comprising a greater proportion of each fragment [8], [9]. Creation of edge habitats results in feedback loops for forest regeneration and fragment viability [10]. Consequently, landscape changes in forest cover and fragmentation can create complex patterns in the distance and intensity of edge penetration for many abiotic and biotic variables, such as ultraviolet light, organismal abundance, and life history patterns [11], [12], [13]. For example, Gehlhausen and colleagues [14] documented edge-related gradients in relative humidity, light, and soil moisture, each of which varied depending on edge aspect and matrix composition. Although much is known about edge effects in temperate forests [15], [16], there are fewer comparable data for tropical forests and even less is known about tropical dry forests.

Edge effects are particularly relevant to the forested regions of Madagascar, where anthropogenic disturbance over the last 50 years has resulted in the loss of half of the original forest cover [17]. Forest conversion is due primarily to slash and burn agriculture and anthropogenic fires used to create cattle pasture [18], [19]. The remaining forest is highly fragmented and prone to extreme edge effects [17]. Of the few in situ studies of edge effects conducted to date on the island, most have documented how plant and animal abundance covary with edge proximity in forest fragments [20], [21], [22], [23]. For example, trees in edge habitats are significantly shorter, have smaller diameters, and lower stem frequencies than trees in interior habitats in eastern humid forests in Madagascar, and these dendrometric variations have been linked to edge avoidance in some lemur species, such as fat-tailed dwarf lemurs [24]. However, there are few data on the biological processes resulting from edge creation, particularly in the island' rare tropical dry forests.

Malagasy dry forests are particularly prone to edge effects due to natural and anthropogenic fires along forest edges [17]. This has resulted in multiple fragments of varying sizes throughout the landscape and along the perimeter of continuous forest tracts. Most significantly, frequent anthropogenic fires suppress forest regeneration, leading to the encroachment of savannah into the fragmented forest and increasing the amount of forest edge that abuts savannah [18], [25]. Moreover, existing data suggest that biotic and abiotic variables are affected up to 400 and 625 meters into the dry forest interior, respectively [26]. These edge penetration distances are much higher than those measured at most other tropical forest sites, where the distance of edge effects rarely exceed 100–125 m [6]. Despite invaluable information on the institutional characteristics influencing patterns of loss and regeneration in Madagascan dry forests [27], there are few data on how edge creation leads to changes in plant biology, such as clinal variations in isotope fractionation for trees along edge-interior gradients.

**Figure 1:**
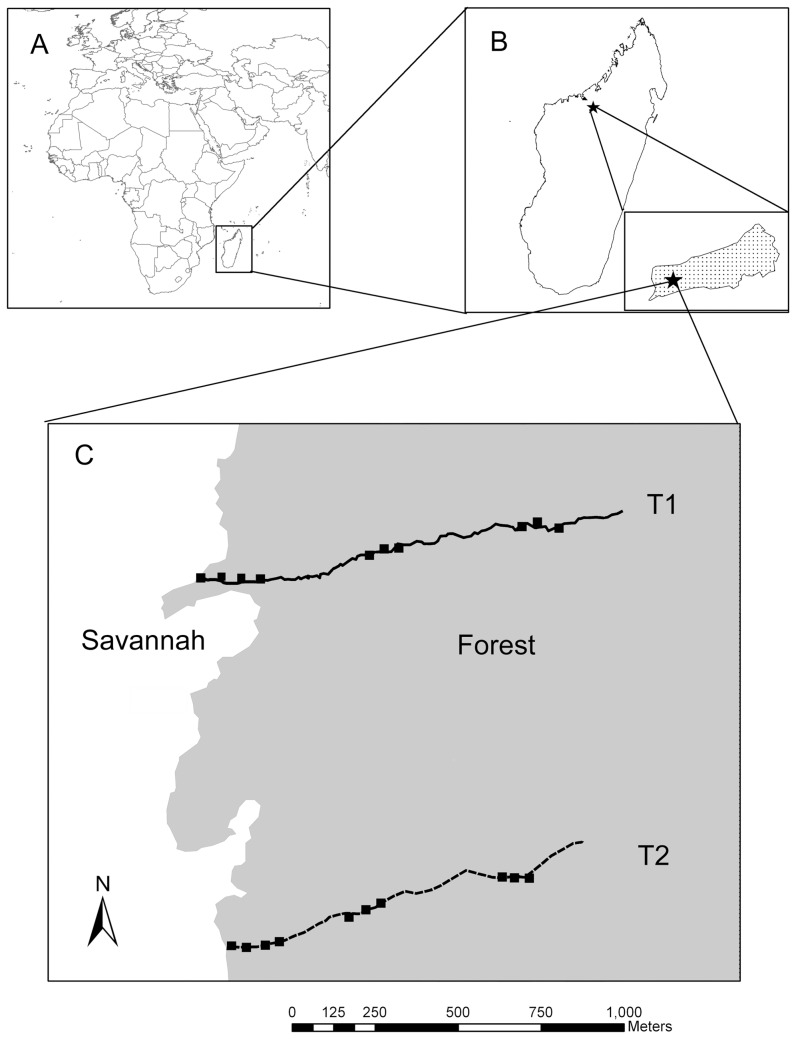
Map of study area at Ampijoroa. Map includes location of Madagascar (A), Ampijoroa Forest Station in Ankarafantsika National Park (B), and Transects and sample plots in our study area along the western border of Ampijoroa (C).

**Figure 2:**
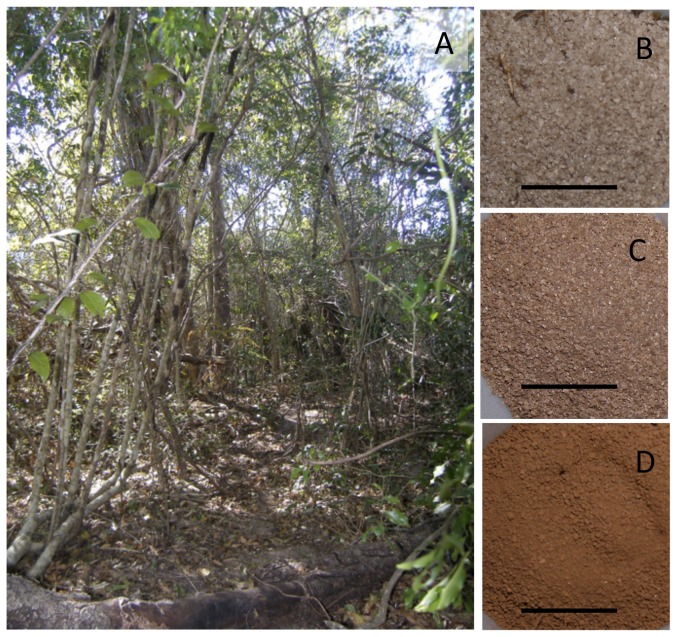
Photographs of the forest interior and examples of soils at Ampijoroa. Images include the dry deciduous forest at Ampijoroa Forest Station, Jardin Botanique A (A), White, course soil (B), speckled, course soil (C), and red, fine soil (D). Scale bar = 1 cm.

Although most researchers measure abiotic and biotic conditions as separate processes, it is increasingly clear that these processes are highly related. Stable isotope values in leaves integrate both abiotic and biotic factors [28], [29]. They may, therefore, quantify edge effects better than conventionally measured variables (e.g., basal area, species richness, wind speed, light levels, air temperature). Previous isotopic applications to forest edge research have been limited to stable carbon isotopes in tropical moist forests [30]. Edge dynamics in dry forests and the influence of forest edges on foliar nitrogen isotope values have yet to be explored. Accordingly, we conducted the first survey of stable carbon and nitrogen isotope values in tree leaves growing along a savannah-dry forest edge. Leaves were collected at Ampijoroa Forest Station, Jardin Botanique A (JBA) Ankarafantsika National Park, northwestern Madagascar (Figs. 1, 2). We tested three hypotheses:

(H1) Vertical stratification in light levels, temperature, and the isotopic composition of CO_2_ available to plants will result in lower carbon isotope values in understory foliage growing close to the forest floor.

(H2) Increasing relative humidity, and decreasing temperature and light levels will result in lower carbon isotope values with increasing distance from the forest edge.

(H3) Increasing soil moisture and decreasing temperature will result in lower nitrogen isotope values with increasing distance from the forest edge.

### Background on isotopes

The natural abundance of stable isotope values in biological materials, such as leaves, is the product of partitioning of isotopes of the same element with differing masses (e.g., ^15^N versus ^14^N; ^13^C versus ^12^C) during chemical and physical reactions. Because the absolute ratio of heavy isotope (^15^N, ^13^C) to light isotope (^14^N, ^12^C) tends to be very small, isotopic abundance is generally referred to using a standardized “δ” notation, where δ = ((R_sample_/R_standard_)−1)*1000, and R = ^13^C/^12^C or ^15^N/^14^N. Values are reported as parts per thousand (per-mil; ‰). The standards for carbon and nitrogen are Pee Dee Belemnite and Air, respectively.

Foliar δ^15^N values are positively correlated with mean annual temperature and negatively correlated with annual rainfall, relative humidity, soil moisture, and soil nutrient content [28], [29], [31], [32], [33], [34], [35]. Foliar δ^13^C values are influenced by temperature, rainfall, relative humidity, sun exposure, soil moisture and water use efficiency [29], [36], [37], [38]. Additionally, understory leaves can exhibit lower δ^13^C values than those in the canopy. This tendency, called the canopy effect, is caused by low light intensities and uptake of soil-respired CO_2_ by understory plants growing close to the forest floor [39], [40].

Based on these trends, we anticipated finding vertical stratification in foliar δ^13^C values at Ampijoroa. We also expected that both δ^13^C and δ^15^N values of understory leaves would be highest close to forest edges, where trees and soils are exposed to more sun, drier air and higher temperatures. We anticipated that both carbon and nitrogen isotope values would decrease within 400–600 m of the savannah border, and that they would remain low and relatively uniform with increasing distance into the forest interior [26]. We predicted that nitrogen isotope values for canopy leaves would show a similar trend. However, because canopy leaves are exposed to abiotic conditions resembling those in the savannah, we expected that δ^13^C values for canopy leaves would be less affected by edges.

## Results

Isotope data, distance from edge, species, tree height, sample height, diameter at breast height for each leaf sample, soil color, and average soil H_2_O% for each plot are presented in Table S1.

### Foliar δ^13^C and Sample Height (H1)

**Figure 3:**
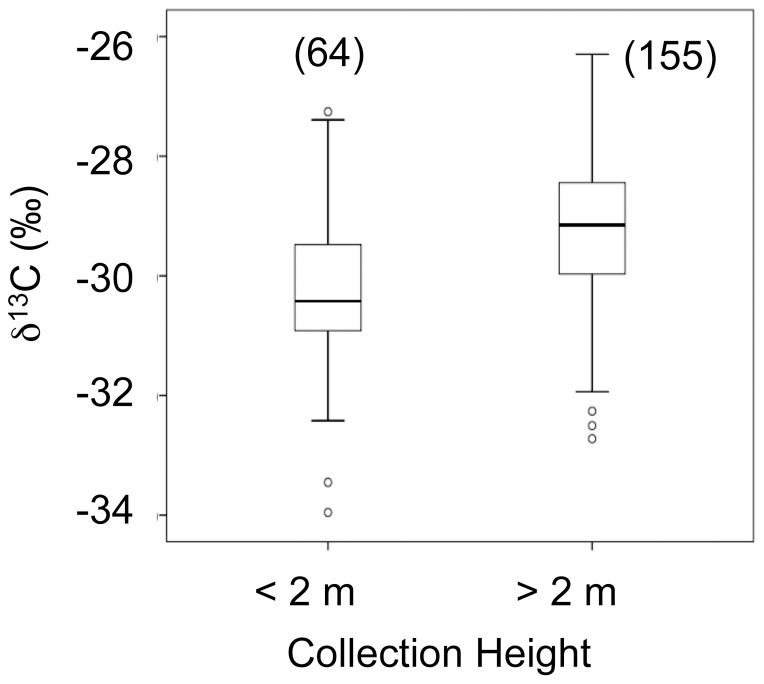
Comparison of foliar δ^13^C levels below and above 2 meters from the ground. “N” values are presented in parentheses. Foliar δ^13^C levels were significantly higher for leaves collected above 2 meters from the ground (F_1,223_ = 36.56, p<0.0001). Removal of outliers did not alter the statistical significance of the test (F_1,217_ = 37.93, p<0.0001).

**Figure 4:**
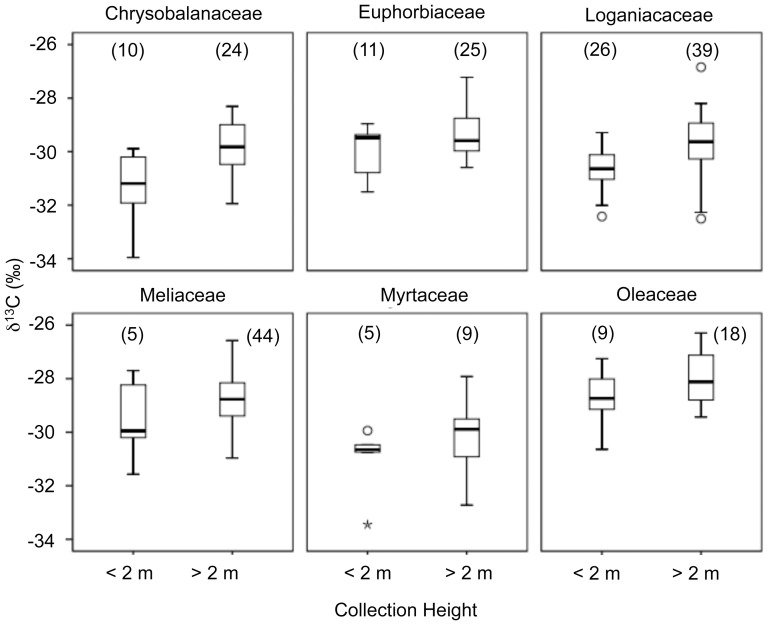
Comparison of foliar δ^13^C values above and below 2 m for by plant family. “N” values are presented in parentheses. Leaves from Chrysobalanaceae (F_1,32_ = 14.33, p = 0.001) and Loganiaceae (F_1,63_ = 16.09, p<0.0001) collected above 2 m had significantly higher δ^13^C values than leaves collected below 2 m. This pattern is marginally significant for Euphorbiaceae (F_1,34_ = 3.88, p = 0.057). There was no significant height effect on foliar δ^13^C values for Meliaceae (F_1,47_ = 1.72, p = 0.196), Myrtaceae (F_1,12_ = 1.34, p = 0.269), or Oleaceae (F_1,25_ = 3.08, p = 0.091). Equality of variances was assessed using Levene's tests. None of the comparisons displayed significant unequal variances (all tests>0.05).

Foliar δ^13^C values were significantly higher in “high" leaves collected above 2 m from the ground compared to “low” leaves sampled below this height threshold (Fig. 3). On average, δ^13^C values for leaves collected below 2 m (−29.2‰) were 1.1‰ lower than leaves collected above 2 m (−30.3‰). This pattern was driven largely by height threshold variations in Chrysobalanaceae and Loganiaceae; none of the other plant families exhibited a significant relationship between height and foliar δ^13^C values (Fig. 4).

### Edge Proximity and Foliar δ^13^C and δ^15^N (H2 & H3)

**Figure 5:**
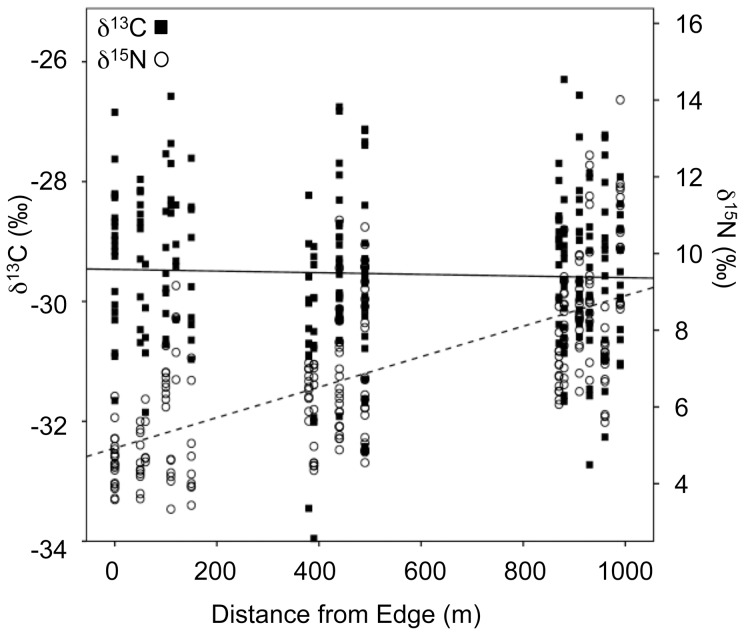
Spatial variability in foliar δ^13^C and δ^15^N values with increasing distance from the forest edge. Carbon isotope values are solid squares and nitrogen isotope values are open circles. The solid regression line is for δ^13^C (F_1,229_ = 0.32, r^2^ = 0.001, p = 0.568) and the dashed line for δ^15^N (F_1,229_ = 194.45, r^2^ = 0.459, p = 0.0001).

**Figure 6:**
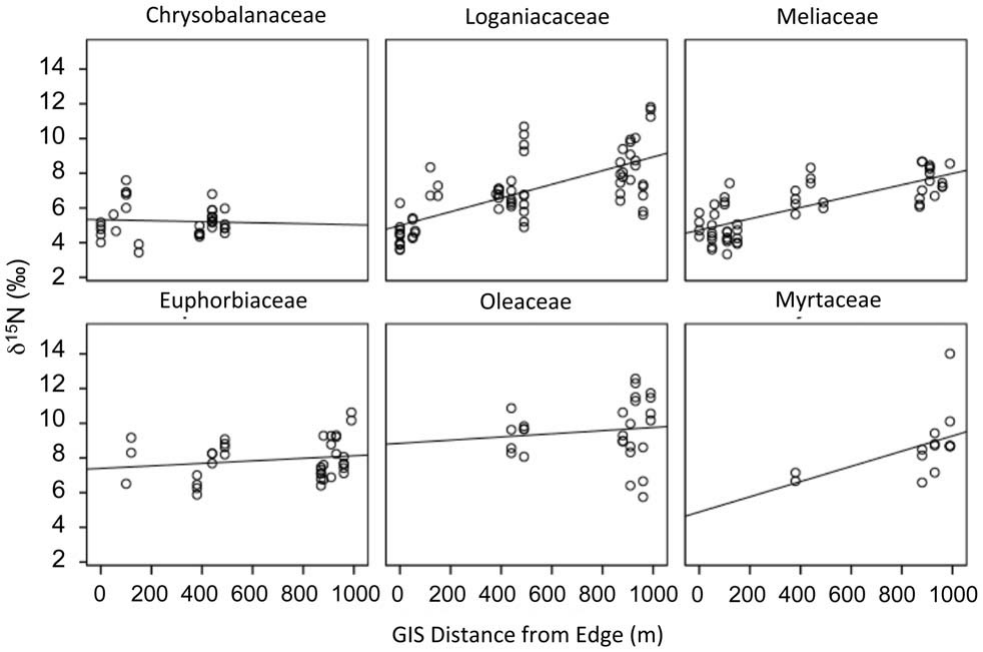
Foliar δ^15^N values and distance from the forest edge by plant family. There is a significant positive relationship between δ^15^N values and distance from the forest edge for Loganiaceae (F_1,65_ = 62.51, r^2^ = 0.489, p<0.0001) and Meliaceae (F_1,50_ = 74.20, r^2^ = 0.597, p<0.0001). Foliar δ^15^N does not covary with edge proximity in Chrysobalanaceae (F_1,33_ = 0.11, r^2^ = 0.003, p = 0.739), Euphorbiaceae (F_1,34_ = 1.16, r^2^ = 0.033, p = 0.288), Myrtaceae (F_1,12_ = 3.83, r^2^ = 0.245, p = 0.072), or Oleaceae (F_1,25_ = 0.34, r^2^ = 0.013, p = 0.564).

Foliar δ^13^C and δ^15^N values differed in their response to edge effects. Foliar δ^13^C did not covary with distance from the forest edge. Conversely, δ^15^N was positively correlated with edge proximity (explaining 46% of the variation in foliar δ^15^N; Fig. 5, Table 1). Of the six plant families from which we collected leaves (Fig. 6), only samples from Loganiaceae and Meliaceae displayed significant correlations between edge distance and δ^15^N.

**Table 1:** Predictors of variability in foliar δ^13^C and δ^15^N values in Generalized Linear Models.

Response Variable	Distance to Edge	Tree Height	Tree DBH	Plant Family	Soil Color	Soil Texture	Soil H_2_O%	2 m Height Threshold	Adjusted r^2^ (%)
δ^13^C	0.152	0.270	0.519	<0.001	0.564	0.994	<0.001	<0.001	44.0
δ^15^N	<0.001	0.071	0.764	<0.001	<0.001	0.462	0.327	0.095	72.9

The p value for each predictor is for a full model that includes all predictors. Significant p values (α = 0.05) are shown in bold. Levene's Test of Equality of Error Variances was not statistically significant for δ^13^C (F = 1.45, p = 0.06) or δ^15^N (F = 1.17, p = 0.25). DBH is diameter at breast height.

### Predictors of Foliar δ^13^C and δ^15^N Variability (H2 & H3)

Three variables (plant family, soil H_2_O%, and 2 m height threshold) were the main predictors of variability in foliar δ^13^C (Table 1). Edge proximity was not a significant determinant of foliar δ^13^C values. The full model explained 44.0% of the total variation in the response variable (F_33,191_ = 14.55, p<0.0001). For δ^15^N, distance from the forest edge, plant family, and soil color were the main drivers of spatial variability (Table 1). Full model performance was relatively stronger than δ^13^C, explaining 72.9% of the variation in δ^15^N (F_33,191_ = 47.28, p<0.0001). Only one predictor variable, plant family, was a significant determinant of spatial variation in both foliar δ^13^C and δ^15^N values.

All multivariate predictors of δ^13^C were statistically significant (Table 2). However, goodness of fit statistics indicate that the best fit model is predicted by plant family and 2 m height threshold, explaining 36.8% of the spatial variation in δ^13^C values. Although the AIC values for the best-fit 2-way model (plant family and height threshold) and 3-way model (plant family, 2 m height threshold, and edge distance) are very similar (65.77 vs. 67.68), the relevant AIC value for the two models, which estimates relative support for a model, indicates that the 3-way model is only 0.384 times as probable as the 2-way model to minimize the information loss (Table 2). For δ^15^N, the clear best-fit model is given by three predictor values (plant family, soil color, and edge distance), which explain 60.8% of the spatial variation in δ^15^N (Table 2).

**Table 2:** Selected multivariate predictors of variability in foliar δ^13^C and δ^15^N values in Generalized Linear Models.

Response Variable	Predictor Variables	Adjusted r^2^	*F*	df	p	AIC
δ^13^C	Family*Edge Distance	0.203	10.74	6	<0.0001	101.4
	Family*Soil Color	0.307	8.28	14	<0.0001	104.9
	Family*Soil H_2_O%	0.272	15.28	6	<0.0001	83.9
	Family*2 m Threshold	0.368	12.86	11	<0.0001	65.7
	Family*2 m Threshold* Edge Distance	0.264	7.70	12	<0.0001	67.6
δ^15^N	Family*Edge Distance	0.559	49.56	6	<0.0001	187.8
	Family*Soil Color	0.661	33.02	14	<0.0001	186.0
	Family*Soil H_2_O%	0.302	17.58	6	<0.0001	304.7
	Family*2 m Threshold	0.448	17.54	11	<0.0001	302.3
	Family*Soil Color*Edge Distance	0.608	29.96	12	<0.0001	103.6

AIC is the Akaike information criterion. AIC-selected best-fit models are indicated in bold.

## Discussion

We predicted vertical stratification in foliar δ^13^C values and decreasing δ^13^C and δ^15^N values with increasing distance from the forest-savannah border. As expected, we observed a canopy effect in δ^13^C values. However, we did not detect the predicted negative relationship between distance from edge and foliar carbon or nitrogen isotope values. Instead, we found no relationship between δ^13^C values and distance from the edge. Quite unexpectedly, we detected a strong positive correlation between distance from edge and δ^15^N values. We address each of these points below.

### H1: Vertical stratification in light levels, temperature, and the isotopic composition of CO_2_ available to plants will result in lower carbon isotope values in understory foliage

On average, δ^13^C values were 1.1‰ lower for leaves collected below 2 meters from the ground. Although small, this difference is significant (Fig. 3). This 1‰ difference is smaller than the canopy effect measured in other dry or deciduous forests [40], [41], a probable reflection of the relatively open nature and short stature of the forest at Ampijoroa (Fig. 2). Additionally, respiration of ^13^C-depleted CO_2_ by soil microbes may be minor at this arid locality.

When broken down by family, the positive relationship between foliar δ^13^C and sample height is only significant for Chrysobalanaceae and Loganiaceae (Fig. 4). However, Euphorbiaceae, Meliaceae, Myrtaceae and Oloeaceae all exhibited a trend towards higher foliar δ^13^C values above 2 meters from the ground (Fig. 4). The varying significance of these trends may simply reflect small sample sizes among the other four families, particularly for samples below 2 m from the ground. Locating leaves close to the ground was difficult for all families except Loganiaceae. Noronhia boiensis tends to be an understory shrub. The other five species sampled are all emergent canopy species.

### Prediction 2: Increasing relative humidity, and decreasing temperature and light levels will result in lower carbon isotope values with increasing distance from the forest edge

We detected no relationship between foliar δ^13^C values and distance from edge, even after accounting for sample height (Tables 1, 2). This is contra the findings of Kapos and colleagues [30], who found a distinctive decrease in the δ^13^C values of understory shrubs with increasing distance from the forest edge. Unlike the Amazonian tropical moist forest where these researchers conducted their study, the forest at Ampijoroa is quite open and the canopy near the edge is relatively discontinuous (Fig. 2). Whereas previous work has detected decreasing wind, light and temperature with increasing distance from the edge [26], these variables may not be consistent enough or large enough to impact foliar δ^13^C values.

### Prediction 3: Increasing soil moisture and decreasing temperature will result in lower nitrogen isotope values with increasing distance from the forest edge

Instead of detecting a negative relationship between δ^15^N and distance from the forest edge, we found a striking positive relationship. We explore five possible explanations for this unexpected trend. First, plant family is a significant predictor of foliar δ^15^N values (Table 2). When broken down by family, only two taxa exhibit a significantly positive relationship, Loganiaceae and Meliaceae (Fig. 6). Despite our efforts to collect leaves from all six families in each of our plots, these two are the only families that are well represented along the entire edge-interior distance. The lack of a relationship between δ^15^N and distance from the forest edge for Chrysobalanaceae, Euphorbiaceae, Myrtaceae and Oleaceae may, therefore, reflect their spatial distribution. None of these families are adequately represented both close to and far from the forest-savannah border.

It is conceivable that interspecific physiological differences are driving the detected relationship between foliar δ^15^N and distance from the edge. Species may differ in their ability to exploit nitrogen sources. Differences in rooting depth, the extent of mycorrhizal associations, and transport, transformation, and reallocation of nutrients within the plant can all influence foliar δ^15^N values [42], [43], [44]. However, such physiological differences should only have small influences on foliar δ^15^N [44], especially considering our attempts to only sample taxa with vesicular-arbuscular mycorrhizas and no N-fixing microbes. It is, therefore, very unlikely that physiological differences among families are responsible for the observed 4.5‰ increase in mean δ^15^N values.

We explore four additional explanations for the unexpected positive relationship between foliar δ^15^N values and distance from the forest edge: (1) anthropogenic activity has disturbed the forest edge, (2) there is less organic input in the forest interior, (3) trees have deeper roots in the forest interior, and (4) soil moisture or nutrient content is lower in the forest interior.

#### Anthropogenic disturbance

The forest at Ampijoroa has a history of human influence [18]. Despite its protected status, there is ample evidence of human disturbance within the forest. While conducting our fieldwork we encountered cows as well as numerous meter-deep pits from locals digging up Dioscorea tubers. Both of these activities disturb the soil and may influence nutrient cycling within the forest [45], but the isotopic ramifications of these impacts are unclear. Based on previous research, we would expect that such disturbances would increase the rate of gaseous ^14^N loss via nitrate oxidation or ammonia volatilization [28], [34], [44]. This should, in turn increase the δ^15^N values of soils and the plants that grow on them. The frequency of cattle and tuber foraging was highest close to the savannah border (Travis Steffens, personal communication), yet this is where we recorded the lowest δ^15^N values. It is possible that this level of human impacts is too low to have an isotopic effect. An alternative possibility is that the entire forest at Ampijoroa can be considered relatively disturbed [46]. Although the forest is currently protected, it is subjected to a steady stream of tourists and scientists. Cattle are also present throughout the reserve. The isotopic effects of human activities near the forest edge may, therefore, be indiscernible.

#### The forest has less organic input than the forest edge

Reduced organic input from leaf litter and decomposition can result in increased soil nitrogen isotope values [35]. Variation in organic inputs might, therefore, be able to explain the observed pattern in δ^15^N. We find this explanation unsatisfactory. Although we have not conducted leaf litter measurements, it seems unlikely that trees growing in the forest interior receive less organic input than those growing closer to the savannah. Ground cover was minimal and leaf litter was thin to absent along the entirety of both transects [47]. Termites living in the savannah could increase plant available-N in the soils. However, their influence should be limited to within 40 m from their mound centers [48]. It is, therefore, highly unlikely that termite-derived nitrogen would be available to forest trees or responsible for the difference in δ^15^N values we have detected between edge and interior leaves.

#### Trees in the forest interior have deeper roots than those growing near the forest edge

If trees growing in the forest interior have deeper roots than those growing closer to the savannah border, they may be accessing a ^15^N-enriched source [28], [42], [49]. Previous research in Ampijoroa has found that trees are shorter and smaller (as measured by diameter at breast height) closer to the savannah [26]. Presumably, smaller plants would have shallower root systems. However, neither tree height nor sample height affects foliar δ^15^N values (Table 2). Thus, while it is certainly possible that interior trees have deep roots, the likelihood that this is driving the observed positive trend between distance from the edge and foliar δ^15^N values is unlikely.

#### Soil moisture or nutrient content in lower in the forest interior

**Figure 7:**
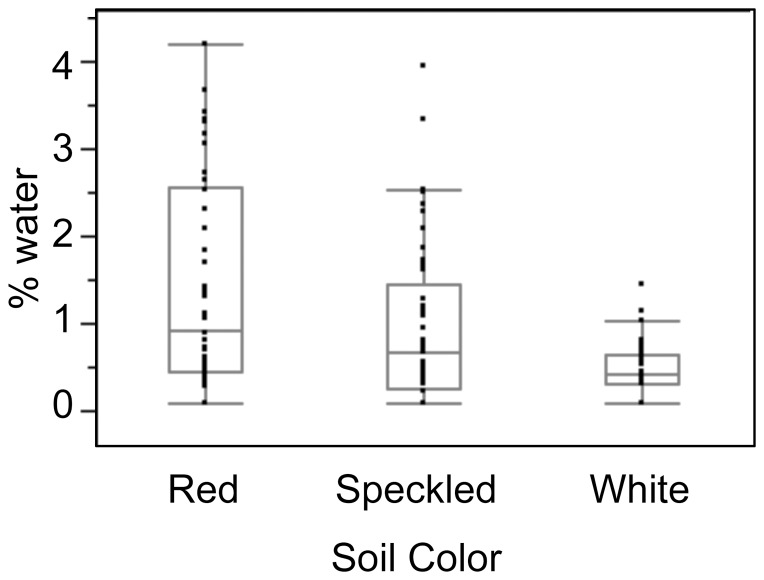
Comparison of water content among soil color categories.

In addition to distance from the edge, δ^15^N covaried with soil color (Table 2). The capacity for soil and plant δ^15^N to vary over small distances is not unexpected. “Microrelief” across seemingly homogenous landscapes can lead to variability both in soil moisture and nutrient retention [31], [50], [51]. The majority of Ampijoroa's precipitation falls during a brief rainy season, which typically occurs from November to April [52]. Red soils near the forest edge appear to retain moisture longer than the more porous white sandy “soils” in the forest interior (Fig. 7). Although all soil samples collected contained very little moisture (<4% by weight), even slight variations in soil moisture could cause trees in the forest interior to experience a more pronounced dry season than those growing closer to the edge. A recent study of island-wide isotopic variability suggests that soil moisture does, indeed, influence plant δ^15^N values [53]. For example, trees growing along the shores of Lake Ravelobe, (<1 km from JBA), exhibit δ^15^N values that are 7‰ lower than those growing in JBA.

Very little research has been conducted on the soils at Ampijoroa. Soil nutrient content has only been measured in three samples of white sandy soils [47]. Nothing is known about the nutrient content of the red soils. Nevertheless, if the soils are as nutrient-poor as suggested by Lourenço and Goodman [47], then the vegetation at Ampijoroa is operating on a very tight nutrient budget. It is, therefore, reasonable to believe that any organic matter that makes its way into the soil is quickly recycled back into the vegetation. During nutrient uptake, plants fractionate against ^15^N. When nutrients are limited, however, plants cannot discriminate as much against ^15^N and they incorporate more of this heavy isotope into their tissues [32]. Additionally, the absence of fine-grained clay minerals in the white sands may make it harder for them to retain labile ions such as nitrate and ammonium [32]. Episodic wetting of the forest soils could lead to the preferential loss of labile ^14^NO_3_^−^ and ^14^NH_4_^+^ via volatilization or leaching [44]. If nutrients are more easily lost from the porous white sands, then interior trees growing on these soils may be recycling their nutrients more completely than those on red soils, a process that would result in increasingly elevated δ^15^N values for plants growing on white sands over time.

Although we were not able to measure nutrient content in the red soils, both their color and finer texture (Fig. 2) suggest that they have more nutrients than the nearly pure quartz white sands. Additionally, in the absence of labile nutrients, trees growing on white sands in the forest interior may be directly accessing nitrogen from organic matter. Reliance on organic nitrogen, particularly free amino acids, is widespread among plants from many ecosystems [54]. Organic N source may differ isotopically from other sources [34]. This is an intriguing possibility that we will investigate in the future.

The positive relationship we have detected between foliar δ^15^N and soil color is opposite to that observed for tropical ‘white sand’ moist forests growing on sandy soils in other parts of the world [49], [50], [55]. Previously, researchers have reported a negative relationship between percent sand and δ^15^N in soils. This difference may reflect the extremely dry nature of Ampijoroa. In addition to growing on nutrient-poor sands, this forest endures very water limited conditions. Mean H_2_O% is only 0.9±1.0%. This idea agrees with previous research in mainland Africa and Australia, where arid, sandy habitats exhibit high δ^15^N values, particularly during the dry season [32], [35], [56], [57].

### Antiquity of the forest-savannah mosaic at Ampijoroa

It is unclear how stable the savannah-forest landscape is in northwestern Madagascar. This landscape may be a stable mosaic ecosystem of relative antiquity [58], [59], [60]. Alternatively, the patchy nature of forest and grasses in and around Ankarafantsika may reflect recently established vegetation responding to frequent anthropogenic disturbance. Arial photographs and vegetation maps indicate that the savannah bordering the forest at Ampijoroa has been present for at least 50 years. However, whether or not it persisted into the deeper past is unknown. Future examination of soil isotope values and radiocarbon ages both in the forest and the savannah will help to clarify the antiquity of the savannahs [59].

The forest at Ampijoroa is a fragile and delicately balanced system. Plants are growing on an extremely limited water and nutrient budget. Humans are clearly affecting the forest here, but the long-term ramifications of their actions are unknown. The savannahs surrounding Ampijoroa have been described as fire-maintained ‘biological deserts’ void of endemic plant species [46], but this may not be entirely accurate. Although frequently burned savannah is dominated by introduced grasses [18], less disturbed savannah contains endemic species [58]. Woody vegetation is generally found on white soils and savannah is generally found on red soils [18]. However, dry forest does grow on red sandy soils (personal observation). Fast-growing savannah grasses likely outcompete trees on these soils following disturbance, such as a brush fire [18]. Conversely, deep-rooted forest vegetation may have a competitive edge over grasses on the white sandy soils. Disentangling anthropogenic and naturally occurring vegetation will have profound implications for conservation. The striking isotopic patterns that we have detected will be useful for understanding foraging patterns in threatened fauna. Continued research on plant responses to edge effects will improve our understanding of the conservation biology of forest ecosystems in Madagascar.

## Materials and Methods

### Site Description

We conducted our fieldwork at Jardin Botanique A (JBA), Ampijoroa Forest Station, Ankarafantsika National Park, northwest Madagascar (Fig. 1). The vegetation at JBA is characterized as semi deciduous dry forest [47]. Canopy height ranges from 5–15 meters and understory and herbaceous vegetation is poorly developed (Fig. 2; [26], [47]). The study area is situated on a sandstone plateau ca. 250 m above see level [46], [61]. The topography is flat, the soil is sandy, acidic and nutrient-poor, and leaf litter is relatively thin and poorly decomposed [47]. The climate at Ankarafantsika is strongly seasonal. Rainfall during most years ranges from 1100–1600 mm per year [52]. The vast majority of this precipitation falls during January and February. Between May and October there is negligible rainfall. Average daily temperatures range from ca. 37°C in the rainy season to 16°C in the dry season [52].

### Sample Collection and Preparation

We carried out our project along the western edge of JBA, where a sharp ecotone divides the forest from the surrounding savannah (Fig. 1). We collected leaf samples along two pre-existing transects which run perpendicular to the savannah edge into the forest interior. Along each transect, we set up 5 m×20 m plots (length perpendicular to transect) at 0, 5, 100, 150, 400, 450, 500, 900, 950, and 1000 m from the savannah-forest boundary (Fig. 1). We recorded geographic coordinates at the center of each plot using a handheld GPS. Using a soil corer, we collected three shallow soil samples (<25 cm) in each plot. We divided soil samples into “top” and “bottom” halves, and recorded their wet weights immediately after collection.

Within each plot, we attempted to collect foliage samples from six tree species representing six plant families (Table 3). These species were chosen because they are food sources for resident Propithecus coquereli groups, and because they are relatively common throughout the study site. All of these families rely on vesicular-arbuscular mycorrhizal (VAM) symbiotic associations [62], [63]. None have symbiotic nitrogen fixing bacteria. We collected 5–10 mature leaves from up to four individuals of each species in each plot. We measured diameter at breast height (DBH) and estimated the height of each individual. Leaves were collected using pruning shears and handheld loppers on a 5-meter extendable pole. In order to control for potential isotopic stratification within the forest, we collected foliage at variable distances above the forest floor (ranging from 0.5–7 m). Whenever possible, we collected both a “low” sample (<2 m) and a “high” sample (>2 m) from the same tree. All specimens were collected between 9 am and 1 pm, July 3 through July 10, 2010.

**Table 3:** Tree species and sample numbers included in this study.

Family	Genus and species	Count
Chrysobalanaceae	Grangeria porosa	35
Euphorbiaceae	Drypetes sp.	37
Loganiaceae	Strychnos madagascariensis	67
Meliaceae	Astrotrichilia asterotricha	53
Myrtaceae	Eugenia tropophylla	14
Oleaceae	Noronhia boiensis	27

After collection, leaf and soil samples were thoroughly air-dried. Soil samples were photographed and their dry mass was recorded. Dried leaves were homogenized using a mortar and pestle. Approximately 5 mg of each homogenized leaf sample were added to tin capsules and combusted on a Finnigan ThermoElectron Delta+XP continuous flow system (Bremen, Germany) connected to a Carlo Erba Elemental Analyzer (Milan, Italy) at the University of California, Santa Cruz Stable Isotope Laboratory. Samples were analyzed for δ^13^C and δ^15^N values, and weight percent carbon, nitrogen, and C/N ratios (here referred to as %C and %N, and C/N). Carbon and nitrogen international standards were Pee Dee Belemnite and Air, respectively. The analytical precision (±1 SD) for carbon was 0.2‰ and 0.08‰ based on 24 IAEA Acetanilide and 23 UCSC oak leaf standard replicates, respectively. Analytical precision for nitrogen was 0.2‰ and 0.1‰ based on Acetanilide and oak replicates. C/N was more variable. Whereas precision based on 24 Acetanilide replicates was 0.1%, precision based on 23 oak replicates was 0.5%. The average difference between 17 leaf samples run in duplicate was 0.1‰ for both δ^13^C and δ^15^N values, and 0.9% for C/N. Raw data are provided in Table S1.

### Analytical Methods

#### Explanatory Variables

We explored how eight explanatory variables covaried with δ^13^C and δ^15^N: (1) distance to edge, (2) tree height, (3) DBH, (4) plant family, (5) soil color, (6) soil grain size, (7) soil moisture content (H_2_O%), and (8) sample height (using a 2 m height threshold for “high” samples above 2 m versus “low” samples below 2 m). Initial comparisons of δ^13^C and distance to edge indicated no significant difference in slopes (t = 1.78, p = 0.09) or y-intercepts (t = 1.86, p = 0.08) between the two transects. There were also no significant inter-transect differences in slopes (t = 0.25, p = 0.80) or y-intercepts (t = 0.72, p = 0.47) for δ^15^N and distance to edge. Therefore, we pooled isotopic data for the two transects. Soil color and grain size were assessed in the field using a relative visual scale. We used two groups for grain size (fine or course) and three groups for color (red, white, or speckled) (Fig. 2). Soil H_2_O% was determined by dividing the dry weight by the wet weight of each sample. There were significant differences in soil H_2_O% among color categories (Kruskal-Wallis X^2^ = 38.03, df = 2, p<0.0001). White sands had the lowest H_2_O% and red sands had the highest (Fig. 7). We arcsine square root transformed proportional data on H_2_O% and log10 transformed tree height and tree DBH. Significance for all tests was set at α = 0.05.

#### Data Analysis

We used an analysis of variance (ANOVA) to test Hypothesis 1 (vertical stratification of biotic and abiotic variables will result in low δ^13^C levels close to the forest floor). Although Levene's test indicated that the population variances were equal for the model (W = 0.017; df = 1,223; p = 0.897), we ran a second test after removing any extreme δ^13^C outliers. We further explored the effects of collection height on δ^13^C levels for each plant family.

We used ordinary least squares regression estimates to test Hypotheses 2 and 3 (that edge proximity is negatively correlated with foliar δ^13^C and δ^15^N values, respectively). One sample Kolmogorov-Smirnov tests indicated that both dependent variables were normally distributed (δ^13^C: K–S = 0.041, df = 231, p = 0.200; δ^15^N: K–S = 0.055, df = 231, p = 0.093). The homoscedasticity of the data further precluded data transformation, for analyses. We also analyzed how nitrogen isotope values varied by distance from the forest edge for each of the six plant families.

We used a model building process with General Linear Models (GLM) to explore the effects of edge proximity and the seven other explanatory variables on spatial variations in δ^13^C and δ^15^N. Multivariate models were based on ecological knowledge of the edge system at Ampijoroa and Akaike Information Criterion (AIC) values for model selection. Soil color, soil grain size, and plant family were recoded as dummy variables. For each dependent variable in full models, we used four factors (soil color, soil grain size, plant family, and 2 m height threshold) and four fixed covariates (distance to edge, tree height, tree DBH, and H_2_O%). The fixed covariates were checked for cross-correlations before being used in the GLMs. Of the six cross-correlations, the strongest correlate was for log10 tree height and log10 tree DBH (r = 0.777, n = 233, p<0.0001). Although the other cross-correlations were statistically significant, none of the Pearson correlation coefficients were greater than 0.08.

## Supporting Information

Table S1 **Plant data.** Isotope data, distance from edge, species, tree height, sample height, diameter at breast height for each leaf sample, and soil color, average soil H_2_O% for each plot.
(XLS)
